# Excision of Portions of the Intestine in the Dog*Reprinted from the “Canada Medical and Surgical Journal,” December, 1883.

**Published:** 1884-07

**Authors:** James Bell

**Affiliations:** Medical Superintendent of the Montreal General Hospital


### Art. XXI.—EXCISION OF PORTIONS OF THE INTESTINE IN THE DOG.*

BY JAMES BELL, M.D.,
Medical Superintendent of the Montreal General Hospital.

[Read before the Canada Medical Association, at Kingston, September, 1883.]

The groundwork of my paper consists of the reports of 14 cases of experimental resections of the intestine in dogs. I was led to make this series of experiments by the observation during the past few years of a number of cases of intussusception, hernial and other strangulations, stricture, ulcer, and the various neoplasms which affect the intestinal canal, which were either subjected to equally severe, but less satisfactory, treatment, or abandoned to die as beyond the reach of surgical aid. When I say "equally severe, but less satisfactory, treatment," I refer of course to the orthodox treatment of intussusception by inflation or even by abdominal section, and endeavoring to replace the inverted bowel; and the production of an artificial anus in hernia when the bowel has sloughed. Emboldened by the recent successes in the various branches of abdominal surgery, I reasoned that, under ordinary circumstances, almost any portion of the intestinal tract—certainly any portion of the small intestine—should be removable with the greatest ease, and if the ends could be kept in close apposition for a sufficient length of time, union should occur. I therefore proceeded to operate upon a number of dogs—although I was warned by veterinary surgeons that the dog was considered by them to be specially susceptible to peritonitis—and I consider the results most satisfactory. The following are brief notes of the cases. I have divided them into two series of seven cases each—the difference being that in the first series catgut sutures only were used to unite the ends of the bowel, and in the second series silk sutures only were used. The operations were all done under carbolic spray :—

*First Series.*

Case I.—A young mongrel terrier bitch, dirty, ill-nourished, and suffering from distemper, was operated on on the 24th of

* Reprinted from the "Canada Medical and Surgical Journal," December, 1883.

March last. An incision about three inches long was made in the median line of the abdomen. The omentum was drawn up out of the way with the finger, and a coil of the small intestine withdrawn through the wound. The several branches of the mesenteric artery leading to this portion of the bowel were ligatured by passing catgut ligatures around each with an aneurism needle. The portion of gut thus isolated, $3\frac{1}{2}$ inches long, was then cut off with the scissors, an assistant holding the ends beyond, between the finger and thumb of each hand, to prevent the escape of contents. I then tried to pare away the peritoneal surface of the upper portion of the bowel, intending to push it into the lower portion after having pared away the mucous surface of it to correspond, but I found this quite impossible, as the end of the bowel contracted slightly as soon as it was cut, and the mucous membrane became everted to the extent of nearly a quarter of an inch. I then united the ends by a continuous " over and over " suture of fine catgut, cleansed the bowel thoroughly, and returned it to the abdominal cavity. I closed the abdominal wound with three silver and several catgut sutures, and covered the wound with iodoform, packing it well into the spaces between the sutures. This and all the subsequent operations were done under ether, and as the details of the operation are essentially the same in all, except in the suturing of the ends of the bowel, it will be unnecessary to repeat them. This dog was given a hypodermic injection of ten minims of Liq.Opii Sed., and this was the only medicinal treatment given. None of the others had any medicinal treatment whatever. It spent the day quietly, and took milk greedily in the evening. Its pulse was rapid, and its temperature a little high throughout. The temperature was carefully taken in all the first series of cases, and shows very little elevation—the normal temperature of the dog being about 102° F., and easily disturbed. A liquid stool occurred at the end of 48 hours, and the further progress of the case was uninterrupted. The " distemper " (as shown chiefly by purulent discharge from the nose) continued, and the animal grew thinner and weaker, and died on the 11th of April, 18 days after operation. At the autopsy, 90 hours after death, the abdominal wound was found to be perfectly united and all the organs healthy. There were a few adhesions of omentum and of one coil of small intestine at the site of operation. The union of the bowel was perfect, but had produced considerable narrowing. This I attribute to the puckering produced by the continuous suture. The specimen is labelled "I."* The death in this case, I believe, was due to the "distemper," as there was no other evident cause.

Case II.—This dog was a healthy, well-nourished young mongrel, with some resemblance to the otter-hound species. When brought up for operation on the 28th of March, I found him gorged with meat. My rule in all the cases was to keep the dog on low diet for about two days before operation, and on a moderate milk diet afterwards. This dog had been fed about an hour before the operation by an over-zealous and too humane servant. I proceeded with the operation, however, and removed three inches of the small intestine in the same manner as in the last case. The ends of the gut were brought together by a double row of catgut sutures. The first row consisted of as many interrupted sutures as could conveniently be inserted, and the second row of a continuous "over and over" suture, catching the spaces between the former. The bowel was returned, and the abdomen closed and dressed as in the other case. Copious vomiting of undigested food occurred half an hour after the operation, and a solid stool was passed about 60 hours afterwards. The abdominal sutures were removed on the ninth day, and the dog continued in perfect health. On the 29th of June I ligated his right carotid artery with catgut. He recovered, without a bad symptom; and on the 26th of July, just 4 months (less three days) after resection of the bowel, I killed him by pithing. The body was well nourished—in fact, quite fat—and there were no adhesions in the peritoneal cavity. The organs were all healthy, and the bowel perfectly united.

Case III.—A healthy, well-nourished mongrel bitch; 12½ inches of intestine were removed in the usual way on the 30th of March, and the ends of the bowel united as in Case II. A similar stool was passed on the third day. There was slight elevation of temperature for two or three days, but otherwise the dog was perfectly well after the first 24 hours. She took milk greedily, was playful and active, and never had a bad symptom throughout. On the 29th of April I gave her ether,

* Portions of bowel, showing the results of operation in each case, were exhibited in connection with the paper.

and cut one of her costal cartilages subcutaneously to observe the process of repair in it. On the 29th of May, two months after the first operation, I gave her ether and bled her to death by cutting the femoral artery across. At the autopsy, the body was found to be well nourished, all the organs healthy, no adhesions in the peritoneal cavity, and the union of the bowel perfect. There was no union of the costal cartilage.

Case IV.—A bright, active young fox terrier; six inches of small intestine were removed on the 3d of April. The ends of the bowel were united with twelve interrupted and a continuous catgut suture. The abdominal sutures were removed on the fifth day, when he was apparently perfectly well in every respect. He continued well, and grew fat, until the 22d, when he had a chill, and became feverish. Symptoms of "distemper" came on, and he gradually grew weaker from day to day, and died on the 18th of May, 45 days after the operation. There was slight prolapse of the omentum after removing the abdominal sutures, but it required no treatment. At the autopsy, all the organs were found to be healthy, and the union of the bowel complete, and without adhesions.

It will be observed that in this specimen there is at one place a deficiency, or rather an absence, of mucous membrane. This is due to the fact that in this case I tried to include in my sutures only the peritoneal and muscular coats when uniting the ends of the gut. I found it so difficult, however, that after doing a small portion of the circumference in this way, I desisted, and did as in all the other cases, catching the whole thickness of the bowel, mucous membrane and all, in my sutures. The deficiency in the mucous membrane corresponds (in extent at least) to the portion of bowel sutured in this way.

Case V.—A young, active, and well-nourished mongrel hound was operated on on the 3d of April. Nine inches of bowel were removed, and the ends united by 32 interrupted catgut sutures. He did well, and on the 7th of April, four days after operation, I removed the abdominal sutures. A liquid stool occurred during the operation. In this and the last case I was unable to determine when the first stools were passed after operation, as I had a number of dogs on hand, and was obliged to keep these two in a room with two others. On the next day I found the abdominal wound open, and a large omental protrusion. On the following day I gave him ether, re-opened the wound completely, and returned the omental hernia. He never was right after this operation, and sank gradually, dying on the 14th, eleven days after the resection, and four days after the operation for the return of the omentum. At the autopsy, there was gaping of the ends of the bowel, escape of contents, and general peritonitis. The bowel was partially united. The abdominal sutures were removed too early (on the fourth day), and I attribute the death of this animal to the violence employed in giving ether a second time and returning the omentum. This, I think, probably partially separated the recently united bowel, allowing escape of fæcal matter into the peritoneal cavity.

Case VI.—A toy Scotch terrier pup, weighing $3\frac{1}{4}$ pounds, was operated on on the 5th April. Five inches of bowel were removed, and the ends united by interrupted catgut sutures. Very fine gut was used, as the bowel was very small. The dog did quite well for 48 hours, but was found dead a few hours later. At the autopsy, the ends of the bowel were found lying about three-quarters of an inch apart, the catgut having given way everywhere, and the intestinal contents in the peritoneal cavity, which was intensely engorged throughout. The result of this case determined me to use silk sutures, though I did one more case with catgut.

Case VII.—A mongrel black and tan, aged. Ten inches of gut were removed on the 8th of April, and the ends united by a double continuous suture. The first round was the ordinary post-mortem-room suture—every stitch inserted from within outwards. I did not like the appearance of this when done, and I put in a second round of "over and over" suture of catgut also. The bowels moved on the second day, and the dog made an uninterrupted recovery. On the 29th I cut one of his costal cartilages subcutaneously, which produced no bad symptoms. On the 3d of June, 56 days after the operation, I killed him by blowing air into the jugular vein. At the autopsy, the organs were all healthy. There was no trace of peritonitis, and the bowel was perfectly united.

*Second Series.*

Case I.—An aged black terrier. Eight inches of bowel were removed on the 20th of April, and the ends sutured by twenty interrupted sutures of fine carbolized spun silk. The bowels were moved the same night, a solid stool passing, and the dog made an uninterrupted recovery. On the 10th of June, 51 days after operation, I killed him with prussic acid. All the organs were healthy, and the body well nourished and fat. There was slight adhesion of two coils of intestine at the point of resection, and the bowel was united perfectly.

Case II.—A young mongrel terrier. Five inches of bowel were removed, and the ends united by fine silk sutures. The dog never rallied, and died 68 hours after the operation. At the autopsy, all the organs were found to be healthy. There was general peritonitis, the peritoneal cavity containing about 3 ounces of grumous fæcal matter. The ends of the bowel were found to be in perfect apposition, and the silk sutures holding firmly, but at a point near the mesenteric attachment a segment of the gut had been overlooked, and no suture applied, hence the escape of its contents and the bad symptoms from the first. The sutures had been lost through slitting up the bowel—in fact, the deficiency was not noticed until the bowel had been opened up.

Case III.—A young mongrel terrier. Seven inches of bowel were removed, and the ends united by 28 interrupted sutures, 15 of silk and 13 of catgut (my supply of silk having given out). Just after the abdominal wound had been closed, a violent fit of vomiting came on, and the abdomen was filled with air, which could be heard escaping through the wound in the bowel. The dog did badly from the first, and died at the end of the fourth day—about 101 hours after the operation—from peritonitis. At the autopsy, all the organs were found to be healthy, and the sutures in position, though slackened, and the ends of the bowel somewhat separated. This specimen was not preserved.

Case IV.—A toy terrier bitch, weighing about 3 pounds, decrepit with old age, and blind from double senile cataract. Ten inches of bowel were removed, and the ends united by 21 interrupted sutures on the 3d of June. The bowels were moved on the second day, and there never was a bad symptom. She died on the 4th of August, 62 days after operation. She showed no signs of disease, and died, I believe, of senility. The bowel was found to be perfectly united, and there were no traces of peritonitis. This specimen was unfortunately neglected for a couple of days, and was destroyed by the great heat then prevailing (Aug. 4th).

CASE V.—A small and young smooth terrier. Ten inches of bowel, including the cæcum, were removed in the ordinary way and the ends united by 20 interrupted sutures of fine spun silk on the 24th of June. On the 27th, a solid stool was passed. There never was a bad symptom throughout. The abdominal sutures were removed on the 14th day, and the dog was killed by pithing on the 25th of July, 32 days after operation. At the autopsy, the organs were all found to be healthy, and the body well nourished. There were a few adhesions around the wound in the bowel, but the union was perfect. This specimen shows the attachment of the small to the large gut.

CASE VI.—A young mongrel black-and-tan bitch. Four and a half inches of small intestine were removed and the ends united by 22 interrupted and a continuous suture of fine spun silk on the 1st of July. On the 3d a semi-solid stool was passed; and on the 8th the abdominal sutures were removed. The dog was lively and well throughout. On the 25th, twenty-four days after operation, I killed him by pithing. All the organs were healthy, and the body well nourished. There were some adhesions between the bowel and the abdominal wall, but the union was perfect. I may note here that although only twenty-eight days had elapsed from the operation, not a trace of the silk sutures could be discovered at the autopsy, and you will observe that the union of the bowel is as complete as in those which survived the operation for months.

CASE VII.—An aged mongrel terrier; 13 inches of duodenum, close to the stomach, were removed on the 8th of July, and the ends united by interrupted sutures of fine spun silk. He did not seem to suffer any inconvenience from the operation, and was well and lively, and took his milk greedily. At the end of the third day I left the city for a few days, and on my return, was disgusted to find that he had escaped. I never saw nor heard of him afterwards, but I have reason to believe that a tender-hearted domestic set him at liberty on the day after I left, not knowing that he had been operated on.

Now to summarize the results of these operations—of the 14 dogs operated upon, 4 died from the effects of operation, but all from preventable causes. The first from the giving way of the catgut sutures too early; the second from meddlesome surgery; the third from careless surgery, and the fourth from an accident which could not have been foreseen. Of the remaining 10, one died of senility 62 days after operation, and two died of "distemper"—one on the 18th and one on the 45th day after operation. Six made perfect and complete recoveries, and did not suffer in nutrition or digestion, nor in any other way, and were killed at periods varying from one to three months after operation. Of the fourteenth we have no record. In the first case only was there any constriction of the bowel, and in no case was there the sign of any considerable peritonitis. In fact, in three cases there was absolutely not an adhesion in the peritoneal cavity, and the autopsy might have been made in good faith without discovering that the bowels had ever been interfered with.

[We do not know who the veterinarians were that informed Dr. Bell that dogs were especially prone to peritonitis; they were certainly not men who had had much practical experience either in canine vivisection or abdominal surgery.

We have certainly performed our share, and seen more than most of our colleagues, and can positively assert that the canine family is remarkably and wonderfully free from a tendency to surgical peritonitis. In this regard we have castrated between three hundred and four hundred bitches, with the loss of but one, and that from carelessness on the part of the person administering the anæsthetic.

We have performed intestinal resection on thirteen cases in dogs, with the loss of but three. Our experience entirely conforms to that of Dr. Bell.

We have removed the spleen in many cases; and once, successfully, an elliptical piece from the stomach. The meninges of the brain are also quite resistant to operate interference.

We have also removed stones and bones that were lodged in the intestine, by exposing those organs by abdominal section, making the cut in a longitudinal direction, and have yet to report a fatal case.—B.]

